# Association Between Tobacco Retailer Density and Smoking Among Adults With Diabetes and Serious Mental Illness in New York State

**DOI:** 10.5888/pcd19.210270

**Published:** 2022-01-06

**Authors:** Amani Alharthy, Akiko S. Hosler, Emily Leckman-Westin, Jamie R. Kammer

**Affiliations:** 1 Department of Epidemiology and Biostatistics, University at Albany School of Public Health, Rensselaer, New York; 2 Department of Epidemiology, Princess Nourah bint Abdulrahman University College of Health and Rehabilitation Sciences, Riyadh, Saudi Arabia; 3 New York State Office of Mental Health, Albany, New York

## Abstract

**Introduction:**

Smoking prevalence is high among adults with comorbid diabetes and serious mental illness. This population is at elevated risk of smoking-related health consequences and premature death. We focused on the community environment and investigated the association between tobacco retailer density and smoking in this population.

**Methods:**

We obtained individual-level data from the 2017 Patient Characteristics Survey, a medical record–based survey of patients served by the public mental health system in New York State. We computed the density of state-authorized tobacco retailers at the 3-digit zip code level.

**Results:**

The data included 19,492 adults (aged ≥18) with comorbid diabetes and serious mental illness. Of these, 55.6% resided in New York City, 53.1% were female, 38.1% were non-Hispanic White, 30.7% were non-Hispanic Black, 25.2% were Hispanic, and 38.1% were smokers, including electronic cigarette users. The density of tobacco retailers (range, 6.1–16.4 per 10,000 population) was positively associated with smoking (odds ratio = 1.05; 95% CI, 1.03–1.07) after adjusting for sex, race or ethnicity, education, employment, health insurance coverage, obesity, and region (New York City vs outside New York City). We observed no interaction between region and tobacco retailer density.

**Conclusion:**

Findings of this study suggest that allocating more smoking cessation resources to zip code areas with a high density of tobacco retailers, especially in rural areas, along with supporting policy change to reduce tobacco retailor density, may mitigate the negative health consequences of smoking among people with comorbid diabetes and serious mental illness.

**Summary**

**What is already know on this topic?**

The prevalence of smoking is 2 to 3 times higher among adults with comorbid diabetes and serious mental illness than it is among the general population. Smoking is associated with tobacco retailer density measured at the census tract level or within a specific distance from home in the general population.

**What is added by this report?**

Using statewide, medical record–based data, we demonstrated for the first time that tobacco retailer density measured at the 3-digit zip code level is independently and positively associated with smoking among adults with comorbid diabetes and serious mental illness.

**What are the implications for public health practice?**

Allocating more smoking cessation resources to zip code areas with a high density of tobacco retailers and supporting policy changes to reduce tobacco retailer density may be viable strategies to mitigate the negative health consequences of smoking among adults with comorbid diabetes and serious mental illness.

## Introduction

Smoking is a well-recognized modifiable health risk factor for all people, especially people with diabetes. Smoking increases insulin resistance and blood glucose concentration (1). Furthermore, smoking exacerbates both macrovascular and microvascular complications and is independently associated with premature death among people with diabetes (2,3).

An important but often overlooked group at elevated risk for smoking-related health consequences is people with comorbid diabetes and serious mental illness (SMI). SMI is a mental, behavioral, or emotional disorder resulting in serious functional impairment; it includes schizophrenia, bipolar disorder, and major depression (4). The prevalence of diabetes (5) and smoking (6) among the population with SMI is 2 to 3 times higher than in the general population. In New York State, the prevalence of diagnosed diabetes exceeds the prevalence of heart disease, stroke, kidney disease, liver disease, and cancer combined (7,8). The life expectancy of people with comorbid diabetes and SMI is 15 to 20 years shorter than in the general population, and the high prevalence of smoking is a major contributor to this phenomenon (5). Evidence suggests that smoking cessation can reduce mortality from diabetes and cardiovascular complications in the population with comorbid diabetes and SMI (9). In New York State, smoking cessation counseling and medication is widely available through the state mental health system, yet approximately 40% of patients with comorbid diabetes and SMI smoke (7,8).

Research suggests that environmental context is an independent determinant of at-risk health behavior among people with comorbid diabetes and SMI (10). In the general population, living in a community with a higher density of tobacco retailers is linked to higher smoking prevalence and a lower likelihood of smoking cessation (11–14). No studies have been conducted to understand the relationship between tobacco retailer density and smoking among the population with comorbid diabetes and SMI. To fill this knowledge gap, we investigated the association between tobacco retailer density and smoking, including e-cigarette use, among adults with comorbid diabetes and SMI in New York State. Findings of this study will help to formulate new strategies to aid smoking cessation efforts in the state.

## Methods

The main data source for this study was the Patient Characteristics Survey (PCS). The PCS is a cross-sectional survey among patients receiving mental health services, conducted every 2 years by the New York State Office of Mental Health. The survey includes clinical, behavioral, and sociodemographic information for all patients served by more than 4,000 programs licensed or funded by the New York State Office of Mental Health during a specified 1-week period. Data are not self-reported by patients. Health care providers use a web application to enter or electronically load data from existing electronic health record systems (7). We used deidentified data from 2017, which were the most recent data available at the time we conducted our analysis, in 2020–2021. In 2017, data were for the week ending October 29, 2017.

We collected location information for authorized tobacco retailers from a list compiled by the New York State Department of Taxation and Finance for 2017 (15). For the computation of tobacco retailer densities, we obtained 2017 population estimates at the zip code level from the US Census Bureau’s American Community Survey 5-Year Data (2015–2019) (16).

### Measures

SMI was measured by the question, “Does the client have a serious mental illness/serious emotional disturbance?” Diabetes and obesity status was assessed by the question, “Does the client have any of these chronic medical conditions?” Diabetes and obesity were 2 of the conditions listed. The outcome variable, smoking status, was assessed by the question, “In the last 12 months, did the client smoke cigarettes, vape, or use tobacco products?” The answer was binary (yes or no), and those who had unknown status were recoded as missing data. The main exposure, density of tobacco retailers, was measured per 10,000 population at the 3-digit zip code level (ie, the first 3 digits of the 5-digit zip code), to be consistent with the 3-digit zip code indicated for a patient’s residence. We classified the patient’s residence by region: in New York City or in New York State outside New York City. Previous literature identified the following variables as having a significant association with smoking status in the population with SMI: sex, race or ethnicity, education level, employment status, health insurance coverage, and obesity (17,18). These variables were entered in the analysis as covariates.

### Data analysis

To be included in the analysis, PCS respondents had to be adults aged 18 or older who had SMI and diabetes and reported their smoking status. We analyzed frequency distributions of sociodemographic characteristics and obesity status, and we used Pearson χ^2^ tests to evaluate statistical differences between smokers and nonsmokers within each variable. We generated multivariable models with binary smoking status as the outcome variable and sociodemographic and obesity variables as covariates. After performing a global Moran *I* test to check spatial autocorrelation, we proceeded with model building. Because New York City is a high population-density area with a distinctive urban built environment, we created 2 models, one with an interaction between region and tobacco retailer density and the other without the interaction. We employed complete case analysis because cases with missing information were few (ranging from 0.1% for sex to 5.9% for education). We used SAS version 9.4 (SAS Institute Inc) for analysis.

### Mapping

We created a map to visualize tobacco retailer density at the 3-digit zip code level. We obtained the shapefile of 3-digit zip code tabulation areas (ZCTAs) from the US Census Bureau (19). We used RColorBrewer, GISTools and cartography packages in R version 4.2.0 (R Foundation for Statistical Computing) to create a choropleth map. We eliminated zip code areas without a population. This study was reviewed and approved by the University at Albany Institutional Review Board.

## Results

PCS data included 175,926 patients; 140,506 were adults aged 18 or older. Of these adults, 132,670 had SMI, 20,277 had diabetes, and 19,702 had comorbid diabetes and SMI. The analytic sample included 19,492 adults with comorbid diabetes and SMI and known smoking status. Of the 19,492 adults, 55.6% resided in New York City, 53.1% were female, 38.1% were non-Hispanic White, 30.7% were non-Hispanic Black, 25.2% were Hispanic, and 38.1% were smokers (Table 1). Most (63.7%) respondents had a middle school or high school education. Most (90.1%) reported that they were unemployed or not in the labor force, but most (95.8%) had health insurance. Smokers and nonsmokers were significantly different (*P* < .001) in all variables. Men, non-Hispanic Black respondents, and those with middle school to high school education were overrepresented among smokers.

**Table 1:** Sociodemographic Characteristics of Adults With Serious Mental Illness and Diabetes, by Smoking Status, Patient Characteristics Survey, New York State, 2017^a^

Variable	Total (N = 19,492)	Smoker (n = 7,417)	Nonsmoker (n = 12,075)
**New York State region served**			
New York City	10,840 (55.6)	3,864 (52.1)	6,976 (57.8)
Outside New York City	8,652 (44.4)	3,553 (47.9)	5,099 (42.2)
**Sex**			
Female	10,347 (53.1)	3,398 (45.8)	6,949 (57.6)
Male	9,135 (46.9)	4,015 (54.2)	5,120 (42.4)
**Race and ethnicity**			
Non-Hispanic Black	5,959 (30.7)	2,720 (36.8)	3,239 (27.0)
Non-Hispanic White	7,389 (38.1)	2,713 (36.7)	4,676 (38.9)
Hispanic	4,891 (25.2)	1,607 (21.7)	3,284 (27.4)
Non-Hispanic multiracial	278 (1.4)	115 (1.6)	163 (1.4)
Non-Hispanic “other”	882 (4.6)	237 (3.2)	645 (5.4)
**Education status**			
Pre-K to fifth grade	532 (2.9)	143 (2.1)	389 (3.5)
Middle school to high school	11,559 (63.7)	4,933 (70.7)	6,626 (59.3)
Some college	2,926 (16.3)	1,094 (15.7)	1,832 (16.4)
College or graduate degree	2,689 (14.8)	687 (9.9)	2,002 (17.9)
Other	433 (2.4)	116 (1.7)	317 (2.8)
**Employment status**			
Employed	1,902 (9.9)	567 (7.8)	1,335 (11.3)
Unemployed/not in labor force	17,238 (90.1)	6,735 (92.2)	10,503 (88.7)
**Health insurance**			
Yes	18,622 (95.8)	7,221 (97.5)	11,401 (94.7)
No	819 (4.2)	184 (2.5)	635 (5.3)
**Obesity**			
Yes	6,858 (35.2)	2,483 (33.5)	4,375 (36.2)
No	12,634 (64.8)	4,934 (66.5)	7,700 (63.8)

a All values are number (percentage) unless otherwise indicated. All values are significantly different between smokers and nonsmokers at *P* < .001; determined by Pearson χ^2^ tests.

We found fifty-one 3-digit zip code areas with a population and 21,258 registered tobacco retailers in New York State in 2017. The density of 3-digit zip code–level tobacco retailers ranged from 6.1 to 16.4 per 10,000 population, with an average density of 10.8 per 10,000 population.

We observed the highest density of tobacco retailers (13–17 per 10,000 population) in parts of New York City, the city of Niagara Falls at the western end of the state, the Mid-Hudson Valley region, where small cities are scattered throughout the rural valley, and the remote northern Adirondack Mountain region (Figure). The lowest density of tobacco retailers (6 to <8 per 10,000 population) tended to cluster in the central and western regions. The global Moran *I* test (*I* = −0.02) of tobacco retailer density indicated no spatially significant clustering or dispersion of tobacco outlet density in the state.

**Figure:**
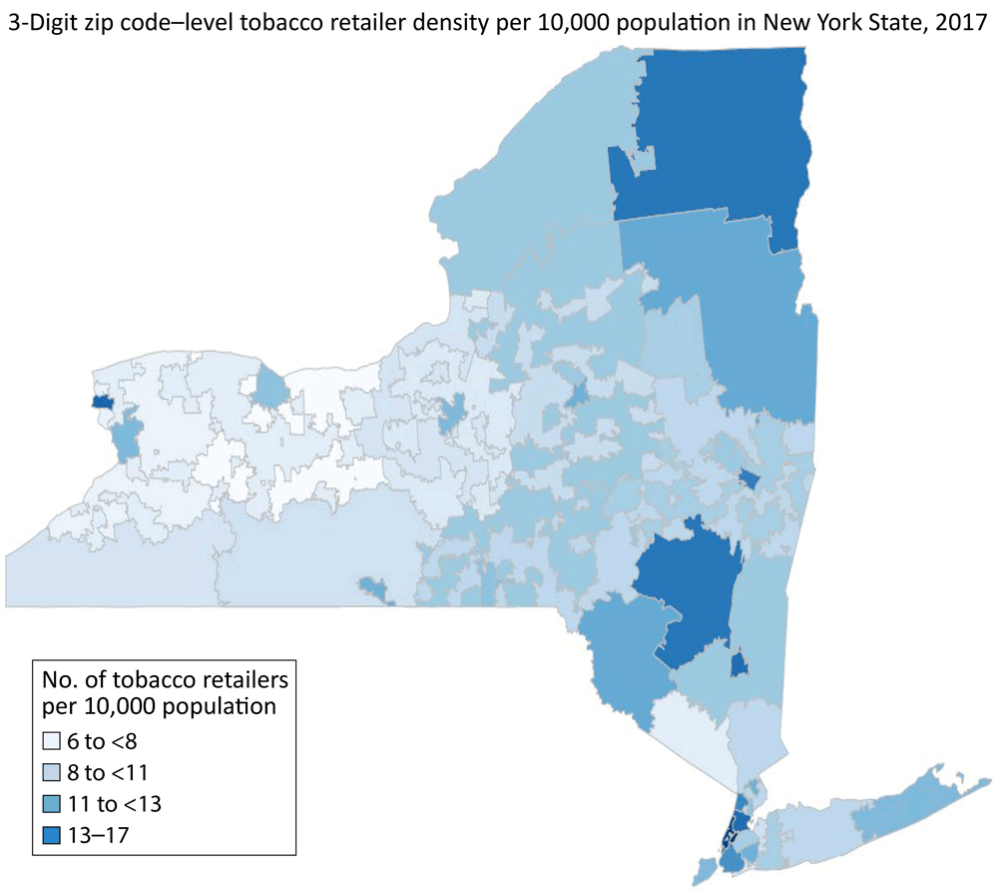
Three-digit zip code–level tobacco retailer density per 10,000 population in New York State, 2017.

Our first model, which included an interaction between tobacco retailer density and region, showed that the density of tobacco retailers was positively associated with smoking (odds ratio [OR] = 1.03; 95% CI, 1.00–1.06) and New York City was negatively associated with smoking (OR = 0.45; 95% CI, 0.30–0.69), but the interaction was not significant (*P* = .14). In the final model, without the interaction, the density of tobacco retailers was positively and independently associated with smoking (OR = 1.05; 95% CI, 1.03–1.07). In addition, all sociodemographic and obesity covariates were significantly associated with smoking in the directions predicted by the bivariate associations with smoking (Table 2).

**Table 2:** Multivariable Logistic Regression Analysis of Smoking Among Adults With Serious Mental Illness and Diabetes, New York State, 2017^a^

Variable	Model 1 odds ratio^b^	Model 2 odds ratio (95% CI)^b^	*P* value
**Density of tobacco retailers**	1.03	1.05 (1.03–1.07)	<.001
**Region**			
Outside New York City	1 [Reference]	1 [Reference]	<.001
New York City	0.45	0.62 (0.56–0.68)	
**Sex**			
Female	1 [Reference]	1 [Reference]	<.001
Male	1.61	1.61 (1.51–1.72)	
**Race and ethnicity**			
Non-Hispanic Black	1.55	1.55 (1.42–1.68)	<.001
Non-Hispanic White	1 [Reference]	1 [Reference]	
Hispanic	0.94	0.94 (0.85–1.03)	.20
Non-Hispanic multiracial	1.34	1.34 (1.03–1.74)	.03
Non-Hispanic “other”	0.76	0.76 (0.63–0.90)	.002
**Education status**			
Pre-K to fifth grade	0.61	0.61 (0.50–0.75)	<.001
Middle to high school	1 [Reference]	1 [Reference]	
Some college	0.84	0.84 (0.77–0.92)	<.001
College or graduate degree	0.50	0.50 (0.45–0.55)	<.001
**Employment status**			
Unemployed/not in labor force	1 [Reference]	1 [Reference]	<.001
Employed	0.65	0.65 (0.58–0.73)	
**Health insurance**			
No	1 [Reference]	1 [Reference]	<.001
Yes	0.40	0.40 (0.33–0.48)	
**Obesity**			
No	1 [Reference]	1 [Reference]	<.001
Yes	0.87	0.87 (0.81–0.93)	

a Data source: Patient Characteristics Survey, 2017 (7).

b Model 1 included an interaction between tobacco retailer density and region; Model 2 did not include the interaction, but instead measured the density of tobacco retailers and its association with smoking.

## Discussion

Adults with diabetes and SMI comorbidity are disproportionately affected by a high prevalence of smoking and its health consequences, including premature death. A high proportion of this group is unemployed, and thus, as a group, they are socioeconomically disadvantaged. Results from our study demonstrate that tobacco retailer density is independently and positively associated with smoking among adults with comorbid diabetes and SMI.

To our knowledge, ours is the first study to focus on a community context to understand smoking behavior in this population. Our study has other strengths. The PCS is a statewide survey that provides data on nearly 20,000 racially and ethnically diverse adults with comorbid diabetes and SMI. This population is hard to reach by conventional survey means such as telephone, mail, or the internet. Information in the PCS data is based on medical records and free of self-reporting bias, such as recall and social desirability biases. Smokers in this study were defined comprehensively and included users of e-cigarettes. The authorized tobacco retailers list is an exhaustive list of tobacco retail outlets in New York State. It accurately reports tobacco retailer locations because it is collected and frequently updated by the government regulator.

Our study also has limitations. The PCS does not provide data on diagnosis and treatment status of SMI, type of diabetes, and whether smokers used tobacco only, e-cigarettes only, or both. The lack of such information did not allow us to establish more specific inclusion criteria. Potential existed for misclassification of diabetes and smoking status. Diabetes is most likely underreported in the PCS: literature suggests as much as 70% of people with SMI are not screened for diabetes (5). The reference period for the smoking question was the past 12 months, not at the time of survey. Potentially important covariates, including physical activity and age categories, are not available in the PCS data. We were not able to assess Asian respondents separately; this group was combined with other non-Hispanic single-race groups in the PCS data. Finally, as a cross-sectional study, temporality of the association between tobacco retailer density and smoking cannot be established.

We should also note that the 3-digit zip code area as a spatial unit for tobacco retailer density measurement is unique. Census tracts are most commonly used in tobacco retailer density research (20-22). It is argued that how researchers define spatial unit — for example, by census tracts, census block groups, or specific distance measures from homes — can influence the measurement of spatial accessibility to tobacco retailers (23). Nonetheless, 3-digit zip code locational information is increasingly used in medical data and in health and environmental research and disease surveillance (24,25). We do not know how well the spatial unit defined by the 3-digit zip code reflects egocentric perception of community, but our study demonstrates that it can be used as a measure of contextual influence on smoking behavior.

Regarding policy implications, legislation to reduce tobacco retailer density has already started in New York State. It primarily focuses on prohibiting sales of tobacco and e-cigarettes in pharmacies. After Rockland County first legislated a ban on tobacco sales in pharmacies in 2017, four more jurisdictions enacted similar legislation: Albany County, Erie County, and New York City in 2018, and Suffolk County in 2019. In our study, Rockland County was the only area in the lowest category of tobacco retailer density in the Downstate region. In May 2020, statewide legislation in New York State ended sales of tobacco and e-cigarette products in all pharmacies in the state.

Pharmacies, however, represent only a small portion of tobacco retailers. Convenience/corner stores and dollar/discount stores are major sources of tobacco and e-cigarettes, and they are overrepresented among newly authorized tobacco retailers, particularly in low-income communities (26). Continuous support for policy changes to reduce the density of tobacco retailers may be a viable strategy; for instance, policy changes could be a moratorium of new tobacco retailer authorization, incentive programs that reward retailers for not selling tobacco or e-cigarettes, and a prohibition of sales of tobacco and e-cigarettes within a certain distance from health facilities and schools. Reducing tobacco retailer density could restrict access to tobacco and e-cigarettes and decrease exposure to harmful point-of-purchase tobacco and e-cigarette advertising among adults and children (27).

As for public health and clinical practice implications, allocating more smoking cessation resources to zip code areas with a high density of tobacco retailers may be feasible. Our study demonstrated that communities with a high density of tobacco retailers exist in both urban and rural settings. Rural communities should be a priority for resource allocation because they have a higher crude prevalence of smoking than urban communities and more barriers to access health care and mental health services (27). Furthermore, exploration is needed into innovative, technology-based smoking cessation programs for rural residents with comorbid diabetes and SMI. Further research to empirically test such interventions is also needed.

Finally, continuous research efforts to monitor the association between the tobacco retail environment and smoking in populations with multiple comorbidities are viable. A multiresolution analysis to incorporate multiple spatial units and analysis of land-area tobacco retailer densities could be used to explore the mechanism of the built environment’s impact on smoking (28). Our research will provide baseline information for future studies to evaluate impacts of changes in tobacco policy and/or smoking cessation intervention on the association.
